# Local Anesthetic Use in Pleural Procedures: Time to Reconsider the Guidelines?

**DOI:** 10.1016/j.chest.2025.04.010

**Published:** 2025-04-18

**Authors:** Craig A. Mounsey, Imogen R. Mechie, Dinesh N. Addala, Rhea Suribhatla, Zara O. Small, Ella M. Smith, Robyn A.E. Gould, Grace A. Annetts, Daniela Krouzkova, Nikolaos I. Kanellakis, Najib M. Rahman

**Affiliations:** a Oxford Pleural Unit, Oxford University Hospitals, Oxford, England; b Chinese Academy of Medical Science Oxford Institute, Nuffield Department of Medicine, Medical Sciences Division, Oxford University, Oxford, England; c Oxford Respiratory Trials Unit, Nuffield Department of Medicine, University of Oxford, Oxford, England; d University Hospitals Bristol NHS Foundation Trust, Bristol, England; e Hampshire Hospitals NHS Foundation Trust, Southampton, England; f Oxford University Medical School, Oxford, England; g Oxford NIHR Biomedical Research Centre, Oxford, England

To the Editor:

Pleural procedures are extremely common, with over 30,000 performed in the United Kingdom every year,^1^ and they are usually undertaken to drain accumulated fluid or air from the pleural cavity for diagnostic or therapeutic purposes.

The skin and parietal pleura are sensitive to pain, and local anesthetic is therefore administered at these points during procedures. Despite this, pain is a common complication of pleural interventions.^2^^,^^3^ In an audit by Hooper et al,^4^ 8% of patients reported pain during chest drain insertion, and 15.6% reported delayed pain after insertion. In an earlier study, 22% of thoracocenteses were complicated by pain.^5^

The British Thoracic Society (BTS) guidelines on local anesthetic use in pleural procedures recommend doses of lidocaine 1% to a maximum of 3 mg/kg (maximum, 250 mg), or 7 mg/kg (maximum, 500 mg) if co-administered with adrenaline.^6^ This is in line with guidelines across most clinical fields.^7^ However, current maximum recommended local anesthetic doses have largely been based on extrapolations from animal experiments and pharmacokinetic studies, rather than controlled trials.^8^ It has been argued that using single blanket maximum doses across patients and procedures is not scientifically valid, because it fails to account for important factors that affect toxicity risk, including renal function, age, comorbidities, injection site vascularity, and the nature of local anesthetic administration.^8^^,^^9^ The BTS guidelines state that there is no consensus on maximum lidocaine dosing in pleural interventions.^6^ Furthermore, although the systemic absorption of lidocaine instilled within the pleural cavity has been investigated,^10^ no studies assessing the absorption of lidocaine administered at the external parietal pleural surface or after pleural procedures have been performed.

In this study, we retrospectively reviewed local anesthetic use and the occurrence of associated adverse events in pleural procedures at Oxford University Hospitals (OUH) over a 2-year period.

## Methods

Data were collected from the procedure reports of all pleural procedures performed at OUH pleural lists between January 2022 and December 2023. Data collected included date of birth, procedure type, and the volume and type of local anesthetic used. Patient sex and weight were collected from electronic medical records, and the doses (in mg/kg) of local anesthetic used in each procedure were calculated. The relationship between patient age or weight and local anesthetic dose in procedures performed without adrenaline co-administration was assessed through Spearman correlation analysis, because Shapiro-Wilk tests demonstrated significant deviations from normality for each variable (*P* < .001).

All patients undergoing pleural procedures in our unit undergo close clinical monitoring for a minimum of 30 minutes after the procedure, during which at least 1 set of further observations is taken. Patient records were also reviewed at least 6 months after a procedure date to assess for evidence of local anesthetic-related adverse events in the periprocedural period.

This study was performed from data retrieved as part of a clinical audit on pleural procedures at OUH, and therefore no formal institutional review board approval was required.

## Results

A total of 930 procedure reports were identified and reviewed. Five hundred seventy-four procedures were conducted in male individuals and 356 in female individuals. Mean age was 69.6 years (range, 17-100 years). The most commonly performed procedure was diagnostic and therapeutic aspiration, followed by image-guided biopsy and chest drain insertion (Table 1). For most procedures, 1% lidocaine alone was used for local anesthesia; however, in indwelling pleural catheter (IPC) insertions and local anesthetic thoracoscopies (LATs), this was typically combined with either bupivacaine 0.5% or lidocaine 1% co-administered with adrenaline.

**Table 1:** Local Anesthetic Use by Pleural Procedure Type

Procedure Type	N	Median Age, y	Most Common Anesthetic Regimen	Mean (SD) Lidocaine Volume, mL	Mean (SD) Lidocaine Dose, mg/kg	Median (IQR) Lidocaine Dose, mg/kg	Lidocaine Dose Range, mg/kg
Dx & Tx aspiration	391	71	Lidocaine 1%	20.3 (4.34)	2.94 (0.85)	2.83 (2.34-3.39)	0.76-6.15
IGBx ± aspiration or ICD insertion	134	75	Lidocaine 1%	21.9 (5.39)	2.98 (0.98)	2.74 (2.30-3.58)	0.91-6.67
ICD insertion	110	72	Lidocaine 1%	21.7 (5.64)	3.23 (0.95)	3.08 (2.62-3.60)	1.58-6.97
Local anesthetic thoracoscopy	95	74	Lidocaine 1% + lidocaine 1%/bupivacaine 0.5% with adrenaline^a^	38.3 (13.6)	4.97 (2.24)	4.51 (3.27-5.88)	1.75-10.75
IPC insertion	89	70	Lidocaine 1% + lidocaine 1% with adrenaline^b^	30.7 (9.80)	4.67 (1.61)	4.25 (3.28-5.96)	2.00-8.75
Dx aspiration only	54	71	Lidocaine 1%	18.7 (4.58)	2.70 (1.07)	2.56 (2.11-3.17)	0.94-7.69
IPC removal	46	76	Lidocaine 1%	21.0 (7.13)	3.17 (1.19)	2.90 (2.50-3.43)	0.49-7.39
Other	10	81	NA	NA	NA	NA	NA

Dx = diagnostic; ICD = intercostal drain; IGBx = image-guided biopsy; IPC = indwelling pleural catheter; IQR = interquartile range; NA = not applicable; Tx = therapeutic.

a Fentanyl and midazolam were used in all procedures as systemic medication. Lidocaine/bupivacaine with adrenaline was used for blunt dissection tract and port entry.

b Lidocaine 1% with adrenaline was used to anesthetize the subcutaneous catheter tract.

**Figure 1:**
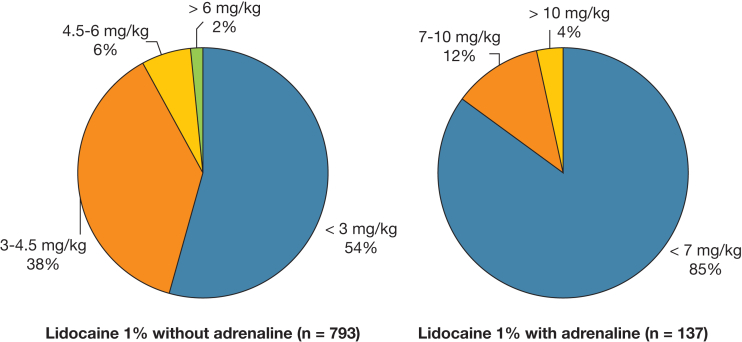
Proportion of pleural procedures with lidocaine doses exceeding typically recommended thresholds.

We sought to identify the number of procedures performed with lidocaine doses exceeding typically recommended thresholds (Fig 1). Of the 793 procedures conducted using lidocaine without adrenaline, 362 (45.6%) used lidocaine at doses > 3 mg/kg, 63 (7.9%) at doses > 4.5 mg/kg, and 13 (1.6%) at doses > 6 mg/kg. In procedures in which adrenaline was also administered (n = 137), 21 (15.3%) used lidocaine at doses > 7 mg/kg and 5 (3.6%) used doses >10 mg/kg. In most procedures, an initial 10 mL 1% lidocaine was administered, with further doses used if the patient appeared to experience pain despite this. However, no formal evaluation of pain was conducted either during or after any procedures.

Strong negative correlation was seen between patient weight and lidocaine dose used (*r* = –0.69; *P* < .001). Age was not significantly correlated with lidocaine dosing (*r* = 0.023; *P* = .52).

On review of postprocedure documentation and observations, no cases of periprocedural local anesthetic-related toxicity were identified.

## Discussion

In this study of a large number of pleural procedures, we have demonstrated that despite doses of local anesthetic frequently exceeding recommended thresholds, and in some cases by a significant margin, there were no occurrences of local anesthetic-related adverse events.

Lower weight was associated with increased lidocaine doses in this cohort, indicating that operators were using typical volumes of local anesthetic across procedures, with limited consideration of dosing. No evidence indicated that operators implemented restricted doses to older patients. A further potential contributing factor to individual patients receiving increased local anesthetic doses is malposition of the initial administration within the pleural cavity rather than at the parietal pleural surface. However, it is standard practice amongst the OUH pleural team to directly visualize local anesthetic administration at the parietal pleura under real-time ultrasound guidance. All procedures were performed with lidocaine at 1% concentration, in line with BTS guidelines advocating dilute preparations with larger volumes to enable spread of the effective anesthetic area. The choice of anesthetic for LATs and IPC insertions was based on institutional-specific preference, rather than direct evidence from studies of pleural procedures, with bupivacaine commonly used in LATs given its longer duration of action compared with lidocaine,^11^ and adrenaline co-administration used to reduce risk of toxicity.

The results of this study have a number of important implications. First, it appears that higher doses of lidocaine than those typically recommended are likely to be safe for many patients undergoing pleural procedures. Second, this work supports previous suggestions that blanket maximum doses for local anesthetics across different patients and procedures are unlikely to be valid. Finally, a number of patients in this study received a combination of different local anesthetic agents, typically lidocaine and bupivacaine. These agents will have an additive effect for toxicity risk, yet current pleural procedural guidelines do not consider the impact of using multiple local anesthetic agents in dosing recommendations.

There are limitations to this study in that it was performed retrospectively, and thus specific scoring systems to detect local anesthetic-related toxicity were not used. During the standardized postprocedure observation period of ≥ 30 minutes, no complications consistent with local anesthetic-related toxicity were demonstrated. However, no studies directly investigating the systemic absorption of local anesthetic after pleural procedures have been performed, and hence it is possible that the mean peak serum concentration falls after the minimum 30-minute observation period. Nonetheless, all patients had at least 1 subsequent episode of care after their pleural procedure available for review on their electronic medical record. We therefore believe it unlikely that any clinically significant occurrences of toxicity were missed.

These initial data strongly suggest that the guideline-referenced limitations on local anesthetic dosing in pleural procedures should be reconsidered, and specific prospective studies conducted to establish tailored pleural intervention-related limits. Maximum doses have historically been recommended to reduce the risk of local anesthetic systemic toxicity, which can be associated with severe cardiovascular and neurologic complications.^12^ However, pain is a frequent occurrence both during and after pleural interventions,^2^, ^3^, ^4^ and limiting the use of local anesthetic can have important implications for patient experience and outcomes. We believe that rather than solely recommending the typical maximum dose, guidelines should reflect the nuance required in these decisions. For instance, while restricting local anesthetic during initial administration to the currently recommended maximum doses is reasonable, if the patient remains in significant pain despite this, it appears from our results that additional local anesthetic is likely to be safe and should be considered. There may be an argument for suggesting a review of patient risk factors for local anesthetic toxicity (such as renal function, cardiac history, and age) before administering these further doses, and 3-lead ECG monitoring may be appropriate in these circumstances. Furthermore, the safety of increased dosing also must be evaluated in the context of the procedural practices and monitoring protocols of individual centers.

## Financial/Nonfinancial Disclosures

None declared.
